# Supplementary material for: A Survey of the Professional Characteristics and Views of Dog Trainers in Canada
Source: Animals (Basel). 2025 Apr 29;15(9):1255. doi: 10.3390/ani15091255 (PMC12070927; doi:10.3390/ani15091255)
Supplement: Supplementary file 1 [file animals-15-01255-s001.zip › animals-3585301-supplementary.pdf]

Supplementary Materials

Survey of Dog Trainers in Canada

CONSENT COVER LETTER

Please check the box at the end of the consent form before proceeding with the survey to indicate that you have read the form.

Title: Survey for dog trainers in Canada

Principal Investigator: Dr. Alexandra Protopopova (a.protopopova@ubc.ca), Animal Welfare Program, Faculty of Land and Food Systems, University of British Columbia.

Co-Investigators: Dr. Camila Cavalli (camila.cavalli@ubc.ca), Animal Welfare Program, Faculty of Land and Food Systems, University of British Columbia & Dr. Sara Dubois, Chief Scientific Officer, British Columbia Society for the Prevention of Cruelty to Animals (BC SPCA).

Introduction: This survey is aimed toward dog trainers who are working professionally (i.e., as a paid occupation) in Canada. This research has the goal of deepening our knowledge about dog trainers in Canada, including the services they offer, their methods, and their beliefs about dogs and dog training. This research is carried out in collaboration between researchers from the Animal Welfare Program at the University of British Columbia and the BC SPCA.

Study Purpose: The purpose of this study is to provide a better understanding of the status of the dog training sector in Canada.

Procedures: Your participation in this study is voluntary. If you agree to participate in this study, you will be asked to complete an online survey that should take you approximately 20 minutes. In this survey, you will be asked questions about yourself and your dog training education, your work as a dog trainer and the types of services you offer, your dog training methods, and your beliefs about dogs and dog training.

Risks and Benefits: There are no expected risks in participating in this study. If at any point you feel uncomfortable, you are encouraged to exit the survey. However, if you exit before finishing the survey, your previous answers may be used for data analysis. While there is no direct personal benefit, your involvement will be helpful to better understand the status of the dog training sector in Canada as well as any emerging animal welfare issues that could be related to it.

Payment: You will not receive payment for participating.

Confidentiality: The privacy and the confidentiality of your personal information will be strictly

protected. We will not collect identifying information (such as your name or your email) at any point. The survey includes some basic demographic questions, such as age, gender, racial origin, and highest level of education. Demographic information is collected to outline which groups are represented (or not) in the sample. You can select the option “prefer not to answer” if you do not feel comfortable sharing that information with the researchers.

Electronic devices used for data collection will be password-protected. All data will be encrypted. Only Drs. Protopopova and Cavalli and relevant co-collaborators within the BC SPCA will have access to the data (for instance, the BC SPCA Science & Policy team).

Research findings may be published or presented at conferences. Results may be published in academic journals, including open-access journals, as well as used for future benchmarking purposes by the BC SPCA. Raw data with no identifying information of participants (as personal identifiers will not be collected nor retained at any stage in this study) may be made publicly available. Providing consent authorizes the release of raw data, which may be used for future research (future use of data will not require consent). As there is no personal identifying information collected in this study, there is no potential for increasing participant risk due to publishing raw data.

Voluntary Participation and Withdrawal: Participation in this study is entirely voluntary, and you have the right to withdraw your consent at any time without penalty or loss of benefits to which you are otherwise entitled. Your decision to participate or withdraw will in no way affect your current or future relationship with the researchers or the institutions. Please note that, as data is collected anonymously, once submitted, you will not be able to withdraw your data.

Funding: This research is funded by the Vancouver Foundation (FO122 – 13648; GR025974).

Contact Information: If you have any questions or concerns about the study, you may contact Dr. Camila Cavalli at camila.cavalli@ubc.ca.

If you have any concerns or complaints about your rights as a research participant and/or your experiences while participating in this study, contact the Research Participant Complaint Line in the UBC Office of Research Ethics at 604-822-8598 or if long distance e-mail RSIL@ors.ubc.ca or call toll free 1-877-822-8598.

Ethics ID H23-03827

By selecting the option below, you are confirming that you have read the consent form and agree to participate in the study:

I consent.

SURVEY

A. The aim for this section is to learn more about you as a dog trainer.

1. How many years have you been training dogs professionally (i.e., as a paid occupation)?

- (Please answer in years) _________

2. Which Canadian province or territory are you located in?

- Alberta
- British Columbia
- Manitoba
- New Brunswick
- Newfoundland
- Northwest Territories
- Nova Scotia
- Nunavut
- Ontario
- Prince Edward Island
- Quebec
- Saskatchewan
- Yukon
- I am not located in Canada

3. What is your age?

- (Please answer in years)_________
- Prefer not to answer

4. What is your gender?

- Man
- Woman
- Non-binary
- Not listed [write in option]
- Prefer not to answer

5. What is your cultural background? Choose all that apply.

- African
- European
- East Asian
- South Asian
- Southeast Asian

- First Nations or Indigenous [please specify]: __
- Hispanic or Latina/Latino
- Middle Eastern
- Other [please specify]: __
- Prefer not to answer

6. Which of the following best describes your highest level of education?

- Some high school
- Completed high school
- Some college/university
- Completed college/university
- Apprenticeship training or trades
- Some graduate education
- Completed graduate education
- Professional degree (e.g., DVM, LLB, MD)
- Other [please specify]: ____
- Prefer not to answer

7. How did you learn how to train dogs? Select the option that best applies to you:

- I am self-educated (e.g., learned by training own dog, reading books, watching training videos, taking continuing education courses)
- I completed one or more training program(s) that provided a certificate upon successful passing (e.g., a series of courses that taught me how to train dogs and assessed my knowledge and skills via exams, written assignments, and/or evaluation of my training)

8. Have you obtained any dog training certifications?

- Yes, from graduating from a training program
- Yes, from taking an exam or evaluation from a credentialing board
- Yes, from both a training program and a credentialing board
- No, I do not have dog training certifications
- Not sure

a) Please write down the name of the training program, or select all that apply from the following list.

- o Absolute Dog Academy: Pro Dog Trainer course
- o Academy for Dog Trainers: Certificate in Training and Counselling (CTC)
- o Animal Behavior College: Animal Behavior College Dog Trainer (ABCDT)
- o Canada West Canine Training: Master Trainer
- o Certified Separation Anxiety Trainer (CSAT)

- o Companion Animal Sciences Institute: Diploma of Canine Behavior Science and Technology (Dip. CBST)
- o Do More With Your Dog: Certified Trick Dog Instructor (CTDI)
- o Education Center: Family Dog Mediator
- o Fear Free: Fear Free Animal Trainer Certification Program
- o Grisha Stewart Academy: Certified BAT Instructor (CBATI)
- o Karen Pryor Academy: Certified Training Partner (KPA-CTP
- o Karen Pryor Academy: Dog Trainer Professional (KPA-DTP)
- o Michael Shikashio: Aggression in Dogs Master Course
- o National Association of Canine Scent Work: Certified Nose Work Instructor (CNWI)
- o Pat Miller/Peaceable Paws: Pat Miller Certified Trainer (PMCT1, PMCT2, PMCT3, or PMCT4)
- o SA Pro: Julie Naismith's Separation Anxiety Pro
- o Victoria Stilwell Academy for Dog Training and Behavior: Victoria Stilwell Academy Dog Trainer Course (VSA-DTC)
- o Other [please specify]:_______________

b) Please write down the certificate(s) you obtained or select all that apply from the following list:

- o Certification Council for Professional Dog Trainers: Certified Professional Dog Trainer-Knowledge Assessed (CPDT-KA)
- o Certification Council for Professional Dog Trainers: Certified Professional Dog Trainer-Knowledge and Skills Assessed (CPDT-KSA)
- o International Association of Animal Behavior Consultants: Certified Behavior Consultant Canine-Knowledge Assessed (CBCC-KA)
- o International Association of Animal Behavior Consultants: Certified Dog Behavior Consultant (IAABC-CDBC)
- o International Association of Animal Behavior Consultants: Certified Animal Behavior
- o Consultant (IAABC-CABC)
- o International Association of Canine Professionals: Certified Dog Trainer (IACP-CDT)
- o Pet Professional Guild: Professional Canine Behavior Consultant (PCBC-A)
- o Pet Professional Guild: Professional Canine Trainer (PCT-A)
- o Other [please specify]: _______________

9. Regulated professions require licenses or certifications from regulatory bodies in order to practice legally. Regulatory bodies set standards for education and professional practice. Dog training is not currently a regulated profession in Canada.

In your opinion, should dog training be a regulated profession in Canada?

- No
- Yes
- Not sure/undecided

10. If dog training were to become a regulated profession, which group(s) do you think should lead and create regulations for dog trainers? Please select all that apply.

- Animal welfare organizations
- Dog training professional organizations (i.e., self-regulation)
- Government agencies
- Other group: ___ [please specify]
- I'm undecided regarding who should lead and create these regulations

B. The aim of this section is to learn more about the dog training services you provide. You will be asked questions related to your dog training and the type of services that you offer.

11. Please tell us how you are currently paid for your dog training services. Select all that apply:

Note: In this context, a business is an activity that you carry out for profit. [Click here] for more information on what a business is.

- I am self-employed with my own dog training business (including a sole proprietorship or a partnership).
- I am employed as a dog trainer by a pet business.
- I am employed as a dog trainer by a shelter or other animal non-profit organization.
- Other [please specify]

12. In which of these ways do you provide dog training? Please select all that apply:

- In-person group classes
- In-person private sessions (working one-on-one with client and dog)
- Online group classes
- Online private sessions
- Self-paced online courses
- Home-based board and train (dog resides in trainer's own home)
- Kennel-based board and train (dog resides in a kenneling facility)
- Training without the owner present ("day training" in which trainer picks up dog from client's home and returns dog after training session)
- Shelter-based training

13. On average, how many hours of training (including preparation time and follow-up) do you carry out each week?

(Please answer in number of hours per week) ___

14. What general types of dog training services do you currently provide? Please select all that apply:

- Basic training (e.g., manners, puppy classes, recall)
- Behavior concerns (e.g., fear, reactivity, aggression)
- Separation anxiety
- Dog sports (e.g., agility, nose work, trick training)
- Service dog training
- Therapy dog training
- Other [please specify]

15. Does the business you do training for also offer any other animal-related services besides dog training? Please select all that apply:

- Animal (pet) boarding (overnight care at a facility)
- Cat training and behavioral support
- Cosmetic dental scaling ("anesthesia-free dental")
- Dog daycare (care during the day at a facility)
- Dog walking
- Pet sitting (care in the pet's home)
- Pet grooming
- Retail pet supplies
- Training for other species [please specify] _____
- Other services: [please specify] ________
- The business I do training for does not offer any other animal-related services

16. What are the most frequent types of training and behavioral concerns that you see in your practice? Please select up to 3 options:

- Bite history toward people
- Bite history toward other dogs
- Destruction/chewing/digging (without separation anxiety)
- Excessive barking (without separation anxiety)
- Fearfulness
- House soiling
- Poor manners (e.g., jumping up, leash pulling, mouthy)
- Poor recall.
- Reactivity toward people
- Reactivity toward other dogs
- Resource guarding
- Separation anxiety
- My current training practice doesn't deal with these concerns
- Other [please specify]

17. Which of the following non-training interventions would you recommend to clients to help with behavioral training? Please select all that apply:

- Acupuncture
- Cannabis products
- Diet—calming
- Diet—low protein
- Diet—raw
- Homeopathy
- Massage therapy
- Pheromones
- Spay/neuter
- Tellington Touch
- Veterinary consultation
- Veterinary consultation specifically to ask for a behavior medication (e.g., Fluoxetine, Gabapentin, Trazodone)
- Veterinary consultation specifically to ask for referral to Veterinary Behaviorist
- I don't make non-training recommendations
- Other: [please specify] ________

C. The aim of this section is to learn more about the characteristics of your training methods:

18. Please select how likely you are to use or recommend the following items

	Extremely likely	Somewhat likely	Neither likely nor unlikely	Somewhat unlikely	Extremely unlikely	I am unfamiliar with this item
Choke collar						
Citronella collar						
Clicker						
Crate						
Electronic collar						
Electronic fence						
Flat collar						
Food rewards/treats						
Harness (back clip)						
Harness (front clip)						
Harness (“no pull”)						

Head collar (e.g., Halti, Gentle Leader)						
Long leash (3 m/9 ft or more)						
Martingale collar						
Muzzle						
Prong collar						
Startling noise (e.g., can with pennies)						
Toys						
Water spray bottle						

19. When you describe your training methods, how likely would it be for you to use the following terms?

	Likely	Neither likely nor unlikely	Unlikely
Balanced			
Dominance theory			
Force-free			
Humane			
Obedience			
Pack leader			
Positive reinforcement			
Relationship-based			
Reward-based			
Science-based			

20. If you would like, please share any other terms you use to describe your training.

21. Please use this space if you have any other comments to share about dog training and trainers in Canada; otherwise, skip this question.

Supplementary Text

Note for Table S1. Training programs identified by 1% or more of respondents as a follow-up to question “Have you obtained any dog training certifications

Full list of credentials mentioned as write-ins (training program names have been preserved as written by the respondents, typos have been corrected when noted, and identical programs have been grouped):

Credentials mentioned 6 times: Leerburg Academy (Michael Ellis courses).

Credentials mentioned 4 times: Canine Academy Canine Obedience Training & Canine Behaviour Therapy Diploma, École de Formation d’Intervenants Canins du Québec (ÉFICQ), Family Paws Parent Education, International Association of Canine Professionals (IACP), International Academy of Canine Trainers, NePoPo, Positive Approach Canine Education through the Cochrane Humane Society (PACE), Shield K9.

Credentials mentioned 3 times: Ben Kersen Professional Trainer Program, Canine Correspondence Studies (CSS) with Norma Jeanne, CATCH Training Academy, Certificate in Applied Animal Behavior from University of Washington, Dognostics, Ian Dunbar’s Top Dog Academy/Ian Dunbar’s Sirius Dog Trainer Academy, ICS Canada—Dog Trainer, International School of Canine Psychology, Ivan Balabanov Training Without Conflict, Superior Canine Course for Behavior Modification Dog Trainers, Tom Rose School, Volhard Motivational Training Camps, Zooacademie Jacinthe Bouchard.

Credentials mentioned 2 times: Absolute Dogs Geek Program, Best Friends Dog Training Certified Pet Dog Trainer (Kim Cooper), Brenda Aloff Course in Training/Brenda Aloff Dog Language and Dog Aggression, Canada West Canine Centre, Canadian Association of Professional Dog Trainers, Canadian Police Canine Association (CPCA), Canine Balance Training by Karen Laws, Control Unleashed Instructor (CCUI), Cooperative Paws–Certified Service Dog Coach, CynoDo Luc Campbell, Edmonton Humane Society Dog Behaviour and Training Methodology Certificate, Denise Fenzi Online Courses, Doggett Style Dog Training, KPA Puppy Start Right Instructor, MSAR Service Dogs, Pat Miller/Peaceable Paws, PetSmart Accreditation, QC Pet Studies, School For Dog Trainers Michael Ellis, School of Canine Science (UK), STSK9 Certified Dog Trainer, TAGteach International, The Naughty Dogge by Monique Anstee, Unleashed Potential Academy.

Credentials mentioned 1 time: Academie Tactique des équipes canines en France by Herve Puppier (France Policier Gendarmerie Royale du Canada), American college of applied science Master’s program, Audeamus Service Dogs Trainer Program, Atlas Assistance Dogs Certified Trainer (Atlas CT), AZCA Formation en comportement animal Quebec, Bark Busters Canada, BC Municipal Police Service Dog Program, Brad Pattison certified trainer educator, B.Sc in Animal Science with a speciality in behaviour and aggression, B.Sc. in Psychology-animal behaviour from Dalhousie University, Calgary Humane Society Apprentice Program, Canadian Canine Search Corps (CCSC), Canadian K9 Dog Trainers Course, Canine Academy—Cambellville Ontario, Canine Complexity Consulting with Cat Harbord, Canine Foundations from Georgian College Barrie, Canine Principles (UK), Canine Strength and Conditioning Coach (CSCC), Certificate in Animal Behaviour and Welfare from University of Edinburgh, Cindy Peacock Dog Training and Behaviour, Cynopraxis Steven R. Lindsay, Dave McMahon Dog Training Academy, Dogue Shop Academy in Montreal QC (ABA1, ABA2, ABA3), Dogstars Animal Training Professional Dog Trainers Program, Dynamic Dog Practitioner, EPICC par Évolution Canine, Formation d’invervenant canin du Québec (FIQ), Highland Canine, Humane Society University online Behavior Assessment Course, International Academy of Canine Trainers (IACT), International Collage of Canine Behavioural Science, International Dog Trainer Education with Turid Rugaa’s,

International Positive Dog Training Association, Institut Belge de Zoothérapie, Jollytails Apprenticeship Program, K9 Academy Apprenticeship, K9 Misfits Mentorship by Bilinda Taras Krupa /Wagner, Karolina Westlund Emotions, Kay Laurence Online Course, L'art au Poils in St-Pie QC (TCAP, CAP, ZAP1), L'académie Chien, Lakeshore Dog Training Assoc, Laura Donaldson's "Slow Thinking is Life Saving for Dogs", Lions Foundation of Canada Dog Guides, Lucky Dog University (Colorado), Mandeville Kennels (Bakersfield CA), M.Sc. in Clinical Animal Behaviour, Master Cyno-professionnel Ethology Institute, McCann Professional Dog Training, National Association of Dog Obedience Instructors (NADOI), Northeast Canine Conditioning (CCAS), Ontario Dog Trainer Academy, Ottawa Canine School, Pima Animal Care Center (PACC), Rainbow paw school (Delicia Maynard), Robert Cabral: Shelter Dog Training, Robin Macfarlane, Roger Abrantes' Ethology Institute Cambridge, Starmark dog training academy, Terry Ryans Chicken camp, The dog wizard, The good dog Sean O'shea shadow program, This Able Veteran–Behesha Doan Certified Service Dog for Veterans, Top Tier K9,Treat-Retreat Certified Suzanne Clothier, TRIBE Training Academy, Tyler Muto: dog reactivity, Unlock Resiliency Course Dr. Kristina Spaulding, West coast canine academy, West Coast Canine Certification, Wizard of Paws–Certified Professional Canine Fitness Instructor, Wunder K9 Apprenticeship program.

Note for Table S2. Training programs identified by 1% or more of respondents as a follow-up to question "Have you obtained any dog training certifications?"

Credentials mentioned 4 times: CKC Canine Good Citizen Evaluator/ CKC Hunt Test Judge, NADOI (National Association of Dog Obedience Instructors).

Credentials mentioned 3 times: BC SPCA AnimalKind, Family Dog Mediator (FDM) L.E.G.S.® Applied Ethology (Kim Brophey), Family Paws Parent Educator, IAABC Certified Animal Behavior Consultant (IAABC-CABC), Regroupement Québécois des Intervenants en Éducation Canine (RQIEC).

Credentials mentioned 2 times: Dynamic Dog Practitioner, IACP-CynoPraxis, Living & Learning with Animals (LLA) Behavior Works (Dr. Susan Friedman), Shield K9, SF SPCA.

Credentials mentioned 1 time: Assistance Dogs International Accredited Program, British Accreditation Council for Canine Behaviour and Psychology, Canadian Association of Professional Dog Trainers, Canadian Police Canine Association, Certification Cyno Professionnelle Canadienne Ltée MCP (Magister Cyno Professionnel), Edmonton Humane Dog Behavior and Training Methodology DBTM, Ford K9, Justice Institute of BC—JIBC Certification for K9 Detection Dogs, IAABC ACDBC, IACT, International College of Canine Behavioural Science, International Positive Dog Training Association IPDTA—CDT, LIMA certified, Pacific Coast K9 Certification for K9 Detection Dog Teams, Sporting Detection Dogs Association, Starmark Training Academy, Tom Rose School Certified Professional Dog Trainer, World Hoopers Association.

Supplementary Table S1

Likelihood of using the following terms to describe their training methods

	Likely	Neither likely nor unlikely	Unlikely	Blank
Balanced	272 (38.52%)	68 (9.63%)	353 (50.00%)	13 (1.84%)
Dominance theory	12 (1.69%)	71 (10.05%)	609 (86.26%)	14 (1.98%)
Force-free	351 (49.71%%)	151 (21.38%)	190 (26.91%)	14 (1.98%)
Humane	590 (83.56%)	82 (11.61%)	21 (2.97%)	13 (1.84%)
Obedience	341 (48.30%)	195 (27.62%)	158 (22.37%)	12 (1.69%)
Pack leader	74 (10.48%)	96 (13.59%)	521 (73.79%)	15 (2.12%)
Positive reinforcement	625 (88.52%)	53 (7.50%)	16 (2.26%)	12 (1.69%)
Relationship-based	571 (80.87%)	90 (12.74%)	33 (4.67%)	12 (1.69%)
Reward-based	606 (85.83%)	73 (10.33%)	15 (2.12%)	12 (1.69%)
Science-based	496 (70.25%)	132 (18.69%)	64 (9.06%)	14 (1.98%)

Supplementary Table S2

Other terms trainers used to describe their training

Terms mentioned	Frequency of mentions
Fear-free	26
Technical terms related to learning theory/conditioning [a]	24
Fun/games-based training	23
Least intrusive minimally aversive (LIMA)	19
Related to “positive balanced” or “positive first” approaches which included the use of aversive methods as a secondary resort	14
Ethical	12
Effectiveness- or results-based	10
Choice and agency; kindness; trust/confidence	9 each
Consent-based; cooperation-based	8 each
Fairness; holistic approaches; welfare	7 each
Dog-centric approaches; working in partnership/as a team with the dog	6 each

Related to “including all 4 quadrants”; leadership/guidance; motivational	5 each
Aversive free; clear/clarity; communication-based; enrichment; respect-based; stress-free/low-stress	4 each
Attachment; calm; coaching; compassionate; engagement; gentle; humane; least inhibitive functionally effective (LIFE); safe	3 each
Adaptive; without pain or intimidation; black and white; client-centered; common sense; connection; cynopraxic; ethological; humane hierarchy; proven; real life/reality; trauma informed; up to date	2 each
Accountability; accommodational; appropriate consequences; avoiding conflict; bonding; cause and effect; commitment; concept based; confident; considerate; consistent; constructional; credentialed/certified; dog-friendly; educational; encouraging; family dog mediator; functional; guidance; hands-on training; human training; incremental; integrative; life changing; life consequences; management; modern; open-minded; personalized; risk-averse; set for success; situation based; stimulation; system based; understanding	1 each

Note. Respondents had the option to share other terms they used to describe their training. These were aggregated using NVivo 14 and are expressed here in decreasing order of frequency. Answers that included terms already presented in question 21 (e.g., “force-free”, “science-based” have been omitted). Responses that mentioned the specific word “balanced” have been omitted in this table, but responses that could be interpreted as balanced training by some people, such as “4 quadrants” or “positive first” have been kept. There were 14 mentions that training methodology should vary depending on the characteristics of each dog, these were not included in the table, as they did not describe specific terms.

[a] Full list of technical terms (some mentioned more than once): capturing, clicker training, exposure, counter conditioning, desensitization, free modeling with reward, luring, negative reinforcement, operant conditioning, progressive reinforcement, proofing, shaping.

Supplementary Table S3

Likelihood of using or recommending the following items

	Extremely likely	Somewhat likely	Neither likely nor unlikely	Somewhat unlikely	Extremely unlikely	I am unfamiliar with this item	Blank
Choke collar	37 (5.24%)	40 (5.66%)	59 (8.35%)	41 (5.80%)	515 (72.94%)	4 (0.56%)	10 (1.41%)
Citronella collar	7 (0.99%)	18 (2.54%)	27 (3.82%)	45 (6.37%)	584 (82.71%)	16 (2.26%)	9 (1.27%)
Clicker	299 (42.35%)	200 (28.32%)	104 (14.73%)	40 (5.66%)	53 (7.50%)	0	10 (1.41%)
Crate	444 (62.88%)	149 (21.10%)	72 (10.19%)	22 (3.11%)	9 (1.27%)	0	10 (1.41%)
Electronic collar	74 (10.48%)	111 (15.72%)	60 (8.49%)	33 (4.67%)	411 (58.21%)	5 (0.70%)	12 (1.69%)
Electronic fence	11 (1.55%)	33 (4.67%)	69 (9.77%)	77 (10.90%)	489 (69.26%)	16 (2.26%)	11 (1.55%)
Flat collar	293 (41.50%)	173 (24.50%)	146 (20.67%)	49 (6.94%)	33 (4.67%)	1 (0.14%)	11 (1.55%)
Food rewards/treats	652 (92.35%)	26 (3.68%)	9 (1.27%)	4 (0.56%)	3 (0.42%)	0	12 (1.69%)
Harness (back clip)	232 (32.86%)	135 (19.12%)	99 (14.02%)	82 (11.61%)	143 (20.25%)	4 (0.56%)	11 (1.55%)
Harness (front clip)	206 (29.17%)	150 (21.24%)	82 (11.61%)	64 (9.06%)	190 (26.91%)	3 (0.42%)	11 (1.55%)
Harness ("no pull")	80 (11.33%)	106 (15.01%)	102 (14.44%)	109 (15.43%)	282 (39.94%)	16 (2.26%)	11 (1.55%)
Head collar (e.g., Halti, Gentle Leader)	57 (8.07%)	163 (23.08%)	115 (16.28%)	157 (22.23%)	199 (28.18%)	3 (0.42%)	12 (1.69%)
Long leash (3 m/9 ft or more)	484 (68.55%)	155 (21.95%)	35 (4.95%)	12 (1.69%)	9 (1.27%)	0	11 (1.55%)
Martingale collar	136 (19.26%)	181 (25.63%)	163 (23.08%)	95 (13.45%)	116 (16.43%)	4 (0.56%)	11 (1.55%)
Muzzle	255 (36.11%)	278 (39.37%)	116 (16.43%)	29 (4.10%)	16 (2.26%)	0	12 (1.69%)
Prong collar	102 (14.44%)	112 (15.86%)	43 (6.09%)	28 (3.96%)	401 (56.79%)	9 (1.27%)	11 (1.55%)
Startling noise (e.g., can with pennies)	27 (3.82%)	50 (7.08%)	62 (8.78%)	66 (9.34%)	480 (67.98%)	8 (1.13%)	13 (1.84%)
Toys	545 (77.19%)	121 (17.13%)	26 (3.68%)	0	3 (0.42%)	0	11 (1.55%)

Water spray bottle	14 (1.98%)	46 (6.51%)	46 (6.51%)	76 (10.76%)	509 (72.09%)	5 (0.70%)	10 (1.41%)

Supplementary Table S4

Associations with provinces

Variables		AB	BC	ON	QC	Chi-Square Test of independence	Fisher's Exact Test
How did you learn how to train dogs?	Completed one or more training programs that provided a certificate	77 (75.75)	160 (156.24)	136 (148.80)	45 (37.20)	X^2 (3, N = 618) = 8.8, *p* = 0.032* Cramer's *V*: 0.119 (weak)	
	Self-educated	35 (36.25)	71 (74.76)	84 (71.20)	10 (17.80)		
	Goodness of fit Chi-square test	X^2 (1, N = 112) = 0.06, p = 0.801	X^2 (1, N = 231) = 0.28, p = 0.597	X^2 (1, N = 220) = 3.4, p = 0.065	X^2 (1, N = 55) = 5.05, p = 0.025* Cramer's *V*: 0.3 (moderate)		
Have you obtained any dog training certifications?	No	23 (28.16)	55 (57.34)	63 (54.55)	12 (12.94)	X^2 (9, N = 603) = 12.13, p = 0.206	
	From credentialing board	22 (17.12)	35 (34.86)	29 (33.16)	7 (7.87)		
	From training program	25 (34.79)	77 (70.84)	69 (67.39)	18 (15.99)		
	From training program + credentialing board	41 (30.93)	59 (62.97)	54 (59.90)	14 (14.21)		
	Goodness of fit Chi-square test	X^2 (3, N = 111) = 8.38, p = 0.039* Cramer's *V*: 0.16 (weak)	X^2 (3, N = 226) = 0.88, p = 0.83	X^2 (3, N = 215) = 2.45, p = 0.484	X^2 (3, N = 51) = 0.42, p = 0.936		
Should dog training be a regulated	Yes	82 (71.11)	143 (147.93)	126 (139.04)	42 (34.92)	X^2 (6, N = 619) = 14.72, p = 0.023*	

profession in Canada?	No		15 (20.99)	45 (43.66)	52 (41.04)	4 (10.31)	Cramer's *V*: 0.11 (Weak)
	Not sure/undecided		15 (19.9)	45 (41.41)	41 (38.92)	9 (9.77)	
	Goodness of fit Chi-square test		X^2 (2, N = 112) = 4.58, p = 0.101	X^2 (2, N = 233) = 0.52, p = 0.772	X^2 (2, N = 219) = 4.26, p = 0.119	X^2 (2, N = 55) = 5.36, p = 0.069	
If dog training were to become a regulated profession, which group(s) do you think should lead and create regulations for dog trainers?	Animal Welfare Organizations	Yes	36 (27.07)	58 (56.1)	44 (53.41)	12 (13.41)	X^2 (3, N = 615) = 6.37, p = 0.095
		No	75 (83.93)	172 (173.9)	175 (165.59)	43 (41.59)	
	Goodness of fit Chi-square test		X^2 (1, N = 111) = 3.89, p = 0.048* Cramer's V: 0.19 (weak)	X^2 (1, N = 230) = 0.09, p = 0.77	X^2 (1, N = 219) = 2.19, p = 0.138	X^2 (1, N = 55) = 0.2, p = 0.657	
	Dog training professional organizations	Yes	62 (58.66)	117 (121.54)	122 (115.73)	24 (29.07)	X^2 (3, N = 615) = 3.36, p = 0.34
		No	49 (52.34)	113 (108.46)	97 (103.27)	31 (25.93)	
	Goodness of fit Chi-square test		X^2 (1, N = 111) = 0.4, p = 0.525	X^2 (1, N = 230) = 0.36, p = 0.548	X^2 (1, N = 219) = 0.72, p = 0.396	X^2 (1, N = 55) = 1.87, p = 0.171	
	Government agencies	Yes	18 (15.34)	29 (31.79)	25 (30.27)	13 (7.6)	X^2 (3, N = 615) = 6.33, p = 0.097
		No	93 (95.66)	201 (198.21)	194 (188.73)	42 (47.4)	
	Goodness of fit Chi-square test		X^2 (1, N = 111) =	X^2 (1, N = 230) =	X^2 (1, N = 219) =	X^2 (1, N = 55) = 4.45, p	

			0.53, p = 0.465	0.28, p = 0.594	1.06, p = 0.302	= 0.035* Cramer's V: 0.28 (moderate)		
When you describe your training methods, how likely would it be for you to use the following terms?	Balanced	Likely	36 (44.43)	76 (89.26)	106 (86.49)	24 (21.82)	X^2 (6, N = 610) = 18.47, p = 0.005* Cramer's V: 0.12 (weak)	
		Not likely nor unlikely	7 (11.57)	28 (23.24)	23 (22.51)	5 (5.68)		
		Unlikely	69 (56)	121 (112.5)	89 (109)	26 (27.5)		
	Goodness of fit Chi-square test		X^2 (2, N = 112) = 6.42, p = 0.04* Cramer's V: 0.17 (weak)	X^2 (2, N = 225) = 3.59, p = 0.166	X^2 (2, N = 218) = 8.08, p = 0.018* Cramer's V: 0.14 (weak)	X^2 (2, N = 55) = 0.38, p = 0.826		
	Dominance-theory	Likely	0 (1.84)	3 (3.69)	4 (3.57)	3 (0.9)	N/A	p = 0.115
		Not likely nor unlikely	11 (11.93)	24 (23.98)	28 (23.23)	2 (5.86)		
		Unlikely	101 (98.23)	198 (197.34)	186 (191.2)	50 (48.24)		
	Goodness of fit Chi-square test		N/A	N/A	N/A	N/A		
	Force-free	Likely	61 (57.1)	113 (114.2)	94 (111.65)	43 (28.04)	X^2 (6, N = 610) = 30.65, p < 0.001* Cramer's V: 0.16 (weak)	
		Not likely nor unlikely	20 (24.05)	60 (48.1)	46 (47.03)	5 (11.81)		
		Unlikely	31 (30.85)	51 (61.69)	79 (60.31)	7 (15.15)		
	Goodness of fit Chi-square test		X^2 (2, N = 112) = 0.95, p = 0.622	X^2 (2, N = 224) = 4.81, p = 0.09	X^2 (2, N = 219) = 8.6, p = 0.014* Cramer's V:	X^2 (2, N = 55) = 16.29, p < 0.001 Cramer's V:		

				0.14 (weak)	0.38 (moderate)		
Humane	Likely	94 (95.14)	189 (191.12)	189 (186.02)	47 (46.72)	N/A	Simulated p-value, b = 2000, p = 0.572
	Not likely nor unlikely	16 (13.38)	25 (26.88)	26 (26.17)	6 (6.57)		
	Unlikely	2 (3.48)	11 (7)	4 (6.81)	2 (1.71)		
Goodness of fit Chi-square test		N/A	X^2 (2, N = 225) = 2.45, p = 0.294	X^2 (2, N = 219) = 1.21, p = 0.547	N/A		
Obedience	Likely	48 (56)	108 (113)	130 (109.5)	20 (27.5)	X^2 (6, N = 612) = 22.54, p = 0.001* Cramer's V: 0.14 (weak)	
	Not likely nor unlikely	33 (31.29)	68 (63.15)	44 (61.19)	26 (15.37)		
	Unlikely	31 (24.71)	50 (49.85)	45 (48.31)	9 (12.13)		
Goodness of fit Chi-square test		X^2 (2, N = 112) = 2.84, p = 0.242	X^2 (2, N = 226) = 0.59, p = 0.743	X^2 (2, N = 219) = 8.89, p = 0.012* Cramer's V: 0.14 (weak)	X^2 (2, N = 55) = 10.21, p = 0.006* Cramer's V: 0.3 (moderate)		
Pack leader	Likely	5 (12.21)	28 (24.64)	29 (24.09)	5 (6.05)	X^2 (6, N = 609) = 19.49, p = 0.003* Cramer's V: 0.13 (weak)	
	Not likely nor unlikely	11 (15.13)	25 (30.53)	43 (29.85)	4 (7.5)		
	Unlikely	95 (83.66)	171 (168.83)	147 (165.06)	46 (41.45)		
Goodness of fit Chi-square test		X^2 (2, N = 111) =	X^2 (2, N = 224) =	X^2 (2, N = 219) =	X^2 (2, N = 55) =		

		6.92, p = 0.031* Cramer's V: 0.18 (weak)	1.49, p = 0.476	8.77, p = 0.012* Cramer's V: 0.14 (weak)	2.31, p = 0.315		
Positive reinforcement	Likely	105 (100.65)	205 (204)	189 (195.92)	51 (49.43)	N/A	p = 0.378
	Not likely nor unlikely	5 (8.97)	16 (18.17)	25 (17.45)	3 (4.4)		
	Unlikely	2 (2.38)	6 (4.82)	4 (4.63)	1 (1.17)		
Goodness of fit Chi-square test		N/A	N/A	N/A	N/A		
Relationship-based	Likely	89 (92.78)	175 (187.23)	195 (181.43)	48 (45.56)	N/A	Simulated p-value, b = 2000, p = 0.032*
	Not likely nor unlikely	17 (13.91)	34 (28.07)	19 (27.2)	6 (6.83)		
	Unlikely	6 (5.31)	17 (10.71)	5 (10.38)	1 (2.61)		
Goodness of fit Chi-square test		X^2 (2, N = 112) = 0.93, p = 0.628	X^2 (2, N = 226) = 5.75, p = 0.056	X^2 (2, N = 219) = 6.27, p = 0.043* Cramer's V: 0.12 (weak)	N/A		
Reward-based	Likely	103 (98.27)	198 (198.3)	191 (192.16)	45 (48.26)	N/A	Simulated p-value, b = 2000, p = 0.34
	Not likely nor unlikely	7 (11.53)	24 (23.26)	25 (22.54)	7 (5.66)		
	Unlikely	2 (2.2)	4 (4.43)	3 (4.29)	3 (1.08)		
Goodness of fit Chi-square test		N/A	N/A	N/A	N/A		

	Science-based	Likely	91 (81.57)	161 (163.87)	149 (159.5)	44 (40.06)	X2(6, N = 611) = 8.99, p = 0.174	
		Not likely nor unlikely	16 (20.16)	42 (40.51)	45 (39.43)	7 (9.9)		
		Unlikely	5 (10.27)	22 (20.62)	25 (20.07)	4 (5.04)		
	Goodness of fit Chi-square test		X^2 (2, N = 112) = 4.65, p = 0.098	X^2 (2, N = 225) = 0.2, p = 0.906	X^2 (2, N = 219) = 2.69, p = 0.261	X^2 (2, N = 55) = 1.45, p = 0.484		
Please select how likely you are to use or recommend the following items	Choke collar	Likely	9 (12.39)	24 (25.23)	30 (24.23)	5 (6.14)	N/A	Simulated p-value, b = 2000, p = 0.038*
		Not likely nor unlikely	9 (9.11)	13 (18.56)	27 (17.82)	1 (4.52)		
		Unlikely	93 (89.49)	189 (182.21)	160 (174.95)	49 (44.34)		
	Goodness of fit Chi-square test		X^2 (2, N = 111) = 1.07, p = 0.586	X^2 (2, N = 226) = 1.98, p = 0.372	X^2 (2, N = 217) = 7.39, p = 0.025* Cramer's V: 0.13 (weak)	N/A		
	Electronic collar	Likely	21 (29.72)	53 (60.52)	82 (58.9)	8 (14.86)	N/A	Simulated p-value, b = 2000, p < 0.001*
		Not likely nor unlikely	14 (9.42)	18 (19.19)	18 (18.68)	2 (4.71)		
		Unlikely	75 (70.86)	153 (144.29)	118 (140.43)	45 (35.43)		
	Goodness of fit Chi-square test		X^2 (2, N = 110) = 5.02, p = 0.081	X2 (2, N = 224) = 1.53, p = 0.464	X2 (2, N = 218) = 12.67, p = 0.002* Cramer's V:	N/A		

					0.17 (weak)			
	Prong collar	Likely	26 (34.29)	61 (70.46)	94 (68.26)	9 (16.99)	N/A	Simulated p-value, b = 2000, p < 0.001*
		Not likely nor unlikely	11 (6.68)	13 (13.72)	13 (13.29)	0 (3.31)		
		Unlikely	72 (68.03)	150 (139.81)	110 (135.45)	45 (33.71)		
	Goodness of fit Chi-square test		X^2 (2, N = 109) = 5.03, p = 0.081	X^2 (2, N = 224) = 2.05, p = 0.359	X^2 (2, N = 217) = 14.49, p = 0.001* Cramer's V: 0.18 (weak)	N/A		
Recommending veterinary consultation	Yes		11 (10.86)	189 (182.19)	154 (175.77)	44 (44.14)	X^2 (3, N = 613) = 27.82, p < 0.001* Cramer's V: 0.27 (Moderate)	
	No		7 (22.11)	38 (44.81)	65 (43.23)	11 (10.86)		
	Goodness of fit Chi-square test		X^2 (1, N = 112) = 12.86, p < 0.001* Cramer's V: 0.27 (Moderate)	X^2 (1, N = 227) = 1.29, p = 0.256	X^2 (1, N = 219) = 13.66, p < 0.001* Cramer's V: 0.27 (Moderate)	X^2 (1, N = 55) = 0, p = 0.961		

Note. *p < 0.05, Cramer's V reported when Chi-square test is significant.

N/A indicates the requirements for the test were not fulfilled.

Supplementary Table S5

Associations with whether dog training should be a regulated profession in Canada

Variables			Yes	No	Undecided	Chi-Square Test of independence	Fisher's Exact Test
How did you learn how to train dogs?	Completed one or more training programs that provided a certificate		331 (289.21)	60 (87.56)	72 (86.23)	X^2 (2, N = 698) = 50.68, p < 0.001*	
	Self-educated		105 (146.79)	72 (44.44)	58 (43.77)	Cramer's *V*: 0.27 (Moderate)	
	Goodness of fit Chi-square test		X^2 (1, N = 436) = 17.94, p < 0.001* Cramer's V: 0.2 (Moderate)	X^2 (1, N = 132) = 25.76, p < 0.001* Cramer's V: 0.44 (Relatively strong)	X^2 (1, N = 130) = 6.98, p = 0.008* Cramer's V: 0.23 (Moderate)		
Have you obtained any dog training certifications?	No		68 (106.12)	59 (31.56)	42 (31.31)	X^2 (6, N = 680) = 73.95, p < 0.001* Cramer's V: 0.23 (Moderate)	
	From credentialing board		83 (70.33)	10 (20.92)	19 (20.75)		
	From training program		125 (130.61)	44 (38.85)	39 (38.54)		
	From training program + credentialing board		41 (30.93)	59 (62.97)	54 (59.90)		
	Goodness of fit Chi-square test		X^2 (3, N = 427) = 24.26, p < 0.001* Cramer's V: 0.14 (weak)	X^2 (3, N = 127) = 43.4, p < 0.001* Cramer's V: 0.34 (Moderate)	X^2 (3, N = 126) = 6.29, p = 0.098		
If dog training were to become a regulated profession, which	Animal welfare organizations	Yes	157 (107.46)	2 31.89)	12 (31.65)	X^2 (2, N = 697) = 83.56, p < 0.001*	
		No	281 (330.54)	128 (98.11)	117 (97.35)		

group(s) do you think should lead and create regulations for dog trainers?	Goodness of fit Chi-square test		X^2 (1, N = 438) = 30.27, p < 0.001* Cramer's V: 0.326 (Moderate)	X^2 (1, N = 130) = 37.13, p < 0.001* Cramer's V: 0.53 (Relatively strong)	X^2 (1, N = 129) = 16.16, p < 0.001* Cramer's V: 0.35 (Moderate)	Cramer's V: 0.35 (Moderate)
	Dog training professional organizations	Yes	61 (68.29)	78 (68.82)	61 (68.29)	X^2 (2, N = 697) = 4.29, p = 0.117
		No	208 (206.12)	52 (61.18)	68 (60.71)	
	Goodness of fit Chi-Square Test		X^2 (1, N = 438) = 0.03, p = 0.857	X^2 (1, N = 130) = 2.6, p = 0.107	X^2 (1, N = 129) = 1.66, p = 0.198	
	Government agencies	Yes	90 (61.58)	4 (18.28)	4 (18.14)	X^2 (2, N = 697) = 41.06, p < 0.001* Cramer's V: 0.24 (Moderate)
		No	348 (376.42)	126 (111.72)	125 (110.86)	
	Goodness of fit Chi-Square Test		X^2 (1, N = 438) = 15.26, p < 0.001* Cramer's V: 0.19 (Weak)	X^2 (1, N = 130) = 12.98, p < 0.001* Cramer's V: 0.32 (Moderate)	X^2 (1, N = 129) = 12.82, p < 0.001* Cramer's V: 0.32 (Moderate)	
When you describe your training methods, how likely would it be for you to use the following terms?	Balanced	Likely	93 (170.74)	105 (51.02)	74 (50.24)	X^2 (4, N = 693) = 191.13, p < 0.001* Cramer's V: 0.37 (Moderate)
		Not likely nor unlikely	39 (42.68)	14 (12.76)	15 (12.56)	
		Unlikely	303 (221.58)	11 (66.22)	39 (65.2)	
	Goodness of fit Chi-Square Test		X^2 (2, N = 435) = 65.63, p < 0.001*	X^2 (2, N = 130) = 103.26,	X^2 (2, N = 128) = 22.24, p < 0.001*	

		Cramer’s V: 0.27 (Moderate)	p < 0.001* Cramer’s V: 0.63 (Strong)	Cramer’s V: 0.29 (Moderate)		
Dominance-theory	Likely	8 (7.54)	3 (2.24)	1 (2.22)	N/A	P < 0.001*
	Not likely nor unlikely	22 (44.63)	28 (13.24)	21 (13.13)		
	Unlikely	405 (382.83)	98 (113.53)	106 (112.65)		
Goodness of fit Chi-Square Test		X^2 (2, N = 435) = 12.79, p = 0.002* Cramer’s V: 0.12 (Weak)	N/A	N/A		
Force-free	Likely	297 (220.64)	14 (65.43)	40 (64.92)	X^2 (4, N = 692) = 196.54, p < 0.001* Cramer’s V: 0.38 (Moderate)	
	Not likely nor unlikely	77 (94.92)	28 (28.15)	46 (27.93)		
	Unlikely	61 (119.44)	87 (35.42)	42 (35.14)		
Goodness of fit Chi-Square Test		X^2 (2, N = 435) = 58.4, p < 0.001* Cramer’s V: 0.26 (Moderate)	X^2 (2, N = 129) = 115.55, p < 0.001* Cramer’s V: 0.67 (Strong)	X^2 (2, N = 128) = 22.6, p < 0.001* Cramer’s V: 0.3 (Moderate)		
Humane	Likely	390 (370.35)	101 (110.68)	99 (108.98)	N/A	Simulated p-value, b = 2000, p < 0.001*
	Not likely nor unlikely	38 (51.47)	22 (15.38)	22 (15.15)		

	Unlikely	7 (13.18)	7 (3.94)	7 (3.88)	
Goodness of fit Chi-Square Test		X^2 (2, N = 435) = 7.47, p = 0.024*, Cramer's V: 0.09 (Negligible)	N/A	N/A	
Obedience	Likely	158 (214.72)	105 (63.38)	78 (62.89)	X^2 (4, N = 694) = 96.47, p < 0.001* Cramer's V: 0.26 (Moderate)
	Not likely nor unlikely	143 (122.79)	18 (36.25)	34 (35.97)	
	Unlikely	136 (99.49)	6 (29.37)	16 (29.14)	
Goodness of fit Chi-Square Test		X^2 (2, N = 437) = 31.71, p < 0.001* Cramer's V: 0.19 (Weak)	X^2 (2, N = 129) = 55.1, p < 0.001* Cramer's V: 0.46 (Relatively strong)	X^2 (2, N = 128) = 9.66, p = 0.008* Cramer's V: 0.19 (Weak)	
Pack-leader	Likely	26 (46.48)	30 (13.81)	18 (13.71)	X^2 (4, N = 691) = 91.04, p < 0.001* Cramer's V: 0.26 (Moderate)
	Not likely nor unlikely	33 (60.3)	39 (17.92)	24 (17.78)	
	Unlikely	375 (327.23)	60 (97.26)	86 (96.51)	
Goodness of fit Chi-Square Test		X^2 (2, N = 434) = 28.35, p < 0.001* Cramer's V: 0.18 (Weak)	X^2 (2, N = 129) = 58.03, p < 0.001* Cramer's V: 0.47 (Relativ	X^2 (2, N = 128) = 4.66, p = 0.097	

			ely strong)			
Positive reinforcement	Likely	414 (393.55)	96 (116.17)	115 (115.27)	N/A	$p < 0.001$
	Not likely nor unlikely	19 (33.37)	24 (9.85)	10 (9.78)		
	Unlikely	4 (10.07)	9 (2.97)	3 (2.95)		
Goodness of fit Chi-Square Test		X^2 (2, N = 437) = 10.92, p = 0.004* Cramer's V: 0.11 (Weak)	N/A	N/A		
Relationship-based	Likely	360 (358.73)	100 (106.14)	111 (106.14)	X^2 (4, N = 694) = 3.74, p = 0.442	
	Not likely nor unlikely	54 (56.54)	22 (16.73)	14 (16.73)		
	Unlikely	22 (20.73)	7 (6.13)	4 (6.13)		
Goodness of fit Chi-Square Test		X^2 (2, N = 436) = 0.2, p = 0.906	X^2 (2, N = 129) = 2.14, p = 0.343	X^2 (2, N = 129) = 1.41, p = 0.494		
Reward-based	Likely	398 (381.59)	100 (112.64)	108 (111.77)	N/A	p = 0.001
	Not likely nor unlikely	34 (45.97)	23 (13.57)	16 (13.46)		
	Unlikely	5 (9.45)	6 (2.79)	4 (2.77)		
Goodness of fit Chi-Square Test		X^2 (2, N = 437) =	N/A	N/A		

			5.91, p = 0.052			
	Science-based	Likely	369 (311.79)	60 (92.46)	67 (91.75)	X^2 (4, N = 692) = 112, p < 0.001* Cramer’s V: 0.28 (Moderate)
		Not likely nor unlikely	52 (82.98)	38 (24.61)	42 (24.42)	
		Unlikely	14 (40.23)	31 (11.93)	19 (11.84)	
	Goodness of fit Chi-Square Test		X^2 (2, N = 435) = 39.16, p < 0.001* Cramer’s V: 0.21 (Moderate)	X^2 (2, N = 129) = 49.17, p < 0.001* Cramer’s V: 0.44 (Relatively strong)	X^2 (2, N = 128) = 23.67, p < 0.001 * Cramer’s V: 0.3 (Moderate)	
Please select how likely you are to use or recommend the following items	Choke collar	Likely	26 (48.51)	33 (14.35)	18 (14.13)	X^2 (4, N = 692) = 96.45, p < 0.001* Cramer’s V: 0.26 (Moderate)
		Not likely nor unlikely	13 (37.17)	24 (11)	22 (10.83)	
		Unlikely	397 (350.31)	72 (103.65)	87 (102.04)	
	Goodness of fit Chi-Square Test		X^2 (2, N = 436) = 32.39, p < 0.001* Cramer’s V: 0.19 (Weak)	X^2 (2, N = 129) = 49.25, p < 0. 001* Cramer’s V: 0.44 (Relatively strong)	X^2 (2, N = 127) = 14.8, p = 0.001* Cramer’s V: 0.24 (Moderate)	
	Electronic collar	Likely	57 (116.26)	90 (34.91)	38 (33.83)	X^2 (4, N = 689) = 266.24, p < 0.001* Cramer’s V: 0.44
		Not likely nor unlikely	9 (37.71)	20 (11.32)	31 (10.97)	

		Unlikely	367 (279.03)	20 (83.77)	57 (81.2)	(Relatively strong)
	Goodness of fit Chi-Square Test		X^2 (2, N = 433) = 79.8, p < 0.001* Cramer's V: 0.3 (Moderate)	X^2 (2, N = 130) = 142.16, p < 0.001* Cramer's V: 0.74 (Strong)	X^2 (2, N = 126) = 44.28, p < 0.001* Cramer's V: 0.42 (Relatively strong)	
	Prong collar	Likely	57 (134.76)	102 (39.93)	55 (39.31)	X^2 (4, N = 686) = 264, p < 0.001* Cramer's V: 0.44 (Relatively strong)
		Not likely nor unlikely	13 (27.08)	12 (8.02)	18 (7.9)	
		Unlikely	362 (270.16)	14 (80.05)	53 (78.8)	
	Goodness of fit Chi-Square Test		X^2 (2, N = 432) = 83.42, p < 0.001* Cramer's V: 0.31 (Moderate)	X^2 (2, N = 128) = 152.95, p < 0.001* Cramer's V: 0.77 (Strong)	X^2 (2, N = 126) = 27.63, p < 0.001* Cramer's V: 0.33 (Moderate)	
Recommending veterinary consultation	Yes		391 (351.31)	69 (104.75)	100 (103.94)	X^2 (2, N = 695) = 86.66, p < 0.001* Cramer's V: 0.27 (Moderate)
	No		45 (84.69)	61 (25.25)	29 (25.06)	
	Goodness of fit Chi-Square Test		X^2 (1, N = 436) = 23.09, p < 0.001* Cramer's V: 0.23 (Moderate)	X^2 (1, N = 130) = 62.81, p < 0.001* Cramer's V: 0.7 (Strong)	X^2 (1, N = 129) = 0.77, p = 0.38	

Note. **p* < 0.05, Cramer's V reported when Chi-Square test is significant.

N/A indicates the requirements for the test were not fulfilled.

Supplementary Table S6

Associations with recommending consultation with a veterinarian

Variables			Yes	No	Chi-Square Test of independence	Fisher's Exact Test
When you describe your training methods, how likely would it be for you to use the following terms?	Balanced	Likely	164 (216.66)	104 (51.34)	X^2 (2, N = 689) = 119.49, p < 0.001 * Cramer's V: 0.42 (Relatively strong)	
		Not likely nor unlikely	54 (54.97)	14 (13.03)		
		Unlikely	339 (285.37)	14 (67.63)		
	Goodness of fit Chi-Square Test		X^2 (2, N = 132) = 96.6, p < 0.001* Cramer's V: 0.14 (Weak)	X^2 (2, N = 557) = 22.89, p < 0.001* Cramer's V: 0.6 (Strong)		
	Dominance-theory	Likely	7 (8.89)	4 (2.11)	N/A	P < 0.001*
		Not likely nor unlikely	36 (56.59)	34 (13.41)		
		Unlikely	514 (491.52)	94 (116.48)		
	Goodness of fit Chi-Square Test		X^2 (2, N = 557) = 8.92, p = 0.012 * Cramer's V: 0.09 (Negligible)	N/A		

Force-free	Likely	329 (283.75)	22 (67.25)	X^2 (2, N = 689) = 93.04, p < 0.001* Cramer's V: 0.37 (Moderate)	
	Not likely nor unlikely	115 (120.45)	34 (28.55)		
	Unlikely	113 (152.79)	76 (36.21)		
Goodness of fit Chi-Square Test		X^2 (2, N = 557) = 17.82, p < 0.001* Cramer's V: 0.13 (Weak)	X^2 (2, N = 132) = 75.21, p < 0.001* Cramer's V: 0.53 (Relatively strong)		
Humane	Likely	480 (472.03)	106 (113.97)	N/A	p = 0.081
	Not likely nor unlikely	59 (66.05)	23 (15.95)		
	Unlikely	16 (16.92)	5 (4.08)		
Goodness of fit Chi-Square Test		X^2 (2, N = 555) = 0.94, p = 0.626	N/A		
Obedience	Likely	233 (273.26)	106 (65.74)	X^2 (2, N = 691) = 60.53, p < 0.001* Cramer's V: 0.3 (Moderate)	
	Not likely nor unlikely	176 (156.38)	18 (37.62)		
	Unlikely	148 (127.36)	10 (30.64)		
Goodness of fit Chi-Square Test		X^2 (2, N = 557) = 11.74, p = 0.003* Cramer's V: 0.1 (Weak)	X^2 (2, N = 134) = 48.79, p < 0.001* Cramer's V: 0.43 (Relatively strong)		

Pack-leader	Likely	32 (58.99)	41 (14.01)	X^2 (2, N = 688) = 95.07, p < 0.001* Cramer's V: 0.37 (Moderate)	
	Not likely nor unlikely	64 (76.77)	31 (18.23)		
	Unlikely	460 (420.23)	60 (99.77)		
Goodness of fit Chi-Square Test		X^2 (2, N = 556) = 18.24, p < 0.001* Cramer's V: 0.13 (Weak)	X^2 (2, N = 132) = 76.83, p < 0.001* Cramer's V: 0.54 (Relatively strong)		
Positive reinforcement	Likely	513 (502.28)	109 (119.72)	N/A	p = 0.003*
	Not likely nor unlikely	35 (42.8)	18 (10.2)		
	Unlikely	10 (12.92)	6 (3.08)		
Goodness of fit Chi-Square Test		X^2 (2, N = 558) = 2.31, p = 0.315	N/A		
Relationship-based	Likely	453 (459.5)	115 (108.5)	X^2 (2, N = 691) = 2.75, p = 0.252	
	Not likely nor unlikely	78 (72.81)	12 (17.19)		
	Unlikely	28 (26.7)	5 (6.3)		
Goodness of fit Chi-Square Test		X^2 (2, N = 559) = 0.53, p = 0.769	X^2 (2, N = 132) = 2.23, p = 0.328		

Reward-based	Likely	499 (486.07)	104 (116.93)	N/A	p = 0.001
	Not likely nor unlikely	46 (58.84)	27 (14.16)		
	Unlikely	12 (12.09)	3 (2.91)		
Goodness of fit Chi-square test		X^2 (2, N = 557) = 3.15, p = 0.207	N/A		
Science-based	Likely	433 (399.45)	62 (95.55)	X^2 (2, N = 689) = 53.52, p < 0.001* Cramer's V: 0.28 (Moderate)	
	Not likely nor unlikely	87 (106.52)	45 (25.48)		
	Unlikely	36 (50.03)	26 (11.97)		
Goodness of fit Chi-square test		X^2 (2, N = 556) = 10.33, p = 0.006 * Cramer's V: 0.1 (Negligible)	X^2 (2, N = 133) = 43.19, p < 0.001 * Cramer's V: 0.4 (Relatively strong)		

Note. **p* < 0.05, Cramer's V reported when Chi-Square test is significant.

N/A indicates the requirements for the test were not fulfilled.

Supplementary Table S7

Themes identified in the "other (write-in)" option for which groups should lead and create regulations for dog training profession

Name of the theme	Description of the theme	Number of occurrences

Accessibility-cost-EDI concerns	Worry about regulation increasing the costs of training and/or creating barriers for systematically excluded groups	9
Against regulation	Against regulation	23
Each dog is different	The individual differences between dogs are too large to have uniform regulations	6
None	None of the organizations proposed in the question (i.e., animal welfare organizations, self-regulation, government agencies) should lead regulation and/or there is no one qualified enough to lead regulation	9
Up to the client	It should be clients who decide who is the best trainer for them	1
Voluntary	Respondent is against mandatory regulation. Each trainer should be free to decide whether to enroll or not	1
Behaviorists	Regulation should be led by behaviorists	2
Collaboration	Specific organisms should collaborate to lead regulation.	35
All of the above welfare + dog training organizations + government	All of the organizations proposed in the question (i.e., animal welfare organizations, self-regulation, government agencies) should take on this role together	6
Consumers/clients		3
Dog training organizations + behaviorists		1
Dog training organizations + government		4
Dog training organizations + researchers		1
Dog training organizations + veterinarians		2
Dog training organizations + welfare organizations		1
Dog training organizations + veterinarians + welfare organizations		1
Researchers-science		1
Stakeholders		1
Welfare organizations + science		2
Welfare organizations + veterinarians		1
Other	This role should fall on organization not mentioned in the other subthemes	1
Creation of a new board	A board should be made up to take on this role	9
Methodology	Respondent indicates how regulation should be implemented/how trainers would prove their proficiency	5
Audited	Trainers should be evaluated by experts in person	1
Exam	Trainers should complete an exam	2

Videos	Trainers should record videos of their training to be evaluated	1
Representation from all methods	Any discussion about regulation should include the views of trainers from different methods (e.g., reward-based as well as balanced trainers)	29
Scientists	Regulation should be led by scientists, researchers, academics, and/or universities	4
Self-regulation	Respondent indicates that regulation should be led by dog training organizations	34
Dog trainers themselves, not whole organizations	Regulation should be led by individual trainers, not organizations	1
Experienced-proven trainers	Regulation should be led by experienced trainers who have proven themselves in some way	15
Only balanced organizations	Regulation should be led by balanced organizations exclusively	1
Only reward-based organizations	Regulation should be led by reward-based organizations exclusively	5
Specific organizations	Respondent indicates organization(s) which should lead regulation	26
Breed groups		1
Canadian Association of Professional Dog Trainers (CADPT)		2
Canadian Kennel Club (CKC)		4
International Association of Animal Behavior Consultants (IAABC)		1
International Association of Canine Professionals (IACP)		8
National Association of Dog Obedience Instructors (NADOI)		2
Pet Professional Guild (PPG)		2
Other	Respondent indicates any organization not included in the other options	1
Service dog organizations		1
Sport groups		4
German Shepherd Schutzhund Club of Canada		1
Specific trainers	Respondent indicates specific trainers/training schools that should lead regulation	3
Michael Shikashio		1
Shield K9 dog training		1
Victoria Stilwell		1
Veterinary Behaviorists	Regulation should be led by veterinary behaviorists	4
Veterinary Organizations	Regulation should be led by veterinary organizations	4
Who should not do it	Respondent indicates organizations which should not lead regulation	29

Dog training organizations	3
Government	9
Veterinarians	1
Welfare organizations	16

Note. Themes based on 163 responses to the “other (write-in)” option. Subthemes were identified when respondent further clarified their position. Totals include mentions of the themes added to mentions of the subthemes.

Supplementary Table S8

Themes identified in the optional “final comments” section

Comments on specific training methods	Respondent expresses their support for specific training practices or indicates concerns about the use of specific training practices including the sentiment that they should be banned. These included the use of reward-based approaches, balanced training, aversive-based tools, and medication. Others highlighted the variability among dogs, expressed in statements such as “not one size that fits all”, and the need to keep an “open mind” and/or “freedom of choice” among differing training approaches	148
Comments about regulation	Respondent mentions the topic of regulation to express agreement/disagreement and/or comment further on it without expressing a clear position. Some of these included concerns about regulation limiting certain types of training, barriers due to cost/accessibility, the difficulty/impossibility of enforcing regulation, and clarification of how regulation should be implemented and/or who should take on the role of leading regulation if it occurred	110
Concerns about the industry	Respondent expresses worry about the dog training industry. Some of the topics mentioned include the lack of trainer education and ease of obtaining online certifications with minimal qualifications, the polarization and disagreement between trainers, the confusion of guardians due to the variability in approaches as well as the use of misleading information, the risks of dogs developing issues due to receiving “bad” training, and concerns about the cost and accessibility of training	90
Comments about the survey	Respondent indicates the survey is biased, not detailed enough, and/or wording is unclear. Respondent comments negatively about the	50

	research team. Respondent expresses concern that the survey could bring negative outcomes to trainers.	
Other comments	Respondent used the space to provide clarification on their survey answers, indicated their appreciation to us for conducting this research, and/or wrote a statement that did not fit in the other themes	28
